# Effects of low doses of the novel dehydroepiandrosterone (DHEA) derivative BNN27 in rat models of anxiety

**DOI:** 10.1007/s00213-023-06490-9

**Published:** 2023-11-02

**Authors:** Evangelia Fragkiadaki, Lamprini Katsanou, Foteini Vartzoka, Achille Gravanis, Nikolaos Pitsikas

**Affiliations:** 1 Department of Pharmacology, Faculty of Medicine, School of Health Sciences, University of Thessaly, Biopolis, Panepistimiou 3, 415-00 Larissa, Greece; 2 Department of Pharmacology, School of Medicine, University of Crete, Heraklion, Greece

**Keywords:** DHEA, BNN27, Anxiety, Rat

## Abstract

**Rationale:**

Several lines of evidence indicate that the neurosteroid dehydroepiandrosterone (DHEA) is involved in anxiety. BNN27 is a new DHEA derivative lacking steroidogenic effects. The beneficial effects exerted by BNN27 in preclinical models of schizophrenia and memory disorders have been recently reported.

**Objectives:**

The present study was designed to investigate the effects of this DHEA novel analog on anxiety-like behavior in rats.

**Methods:**

To this end, the light/dark box, the open field, the contextual fear conditioning, and the excessive self-grooming induced by the serotonin 5-HT_2c_ receptor agonist mCPP tests were utilized.

**Results:**

Animals treated acutely with BNN27 (1, 3, and 6 mg/kg) dose dependently spent more time in the bright compartment of the light/dark box and in the central zone of the open field with respect to their vehicle-treated cohorts. Further, BNN27 reduced freezing behavior and weakened the mCPP-induced excessive self-grooming.

**Conclusions:**

Our data indicate that BNN27 is a highly potent anxiolytic agent, as in all studied paradigms it showed anxiolytic-like effects in male rats.

## Introduction

Anxiety is characterized by a flexible psychological and behavioral status that promote coping when encountered with a potential threat. Anxiety may be turn into a pathological state and interfere with coping. Anxiety disorders comprising generalized anxiety disorder (GAD), phobias, post-traumatic stress disorder (PTSD), and panic disorder are a major public health issue worldwide (Steimer 2002).

So far, molecules acting on the γ-aminobutyric acid (GABA) and serotonergic system, like benzodiazepines, partial agonists of the serotonergic 5-HT_1A_ receptor, and selective serotonin reuptake inhibitors (SSRIs), are widely used for the alleviation of anxiety symptoms. Nonetheless, some forms of anxiety do not respond to pharmacological treatment (Hammer et al. 2004; Van Ameringen et al. 2004).

Further, serious undesired side effects (i.e., sedation, memory impairments, dependence and withdrawal, sexual dysfunction, and increase of body weight) are revealed following treatment with either benzodiazepines or SSRIs. In addition, buspirone, a 5-HT_1A_ receptor partial agonist, although is not associated with appreciable toxicity is scarcely utilized since its efficacy is low (Cryan and Sweeney 2011). Therefore, there is a pressing need for new medications with high efficacy and low toxicity for the treatment of the various forms of the anxiety disorders (Gorman 2003).

It is well documented that steroid hormones exert a regulatory action in growth maturation and differentiation of the brain. Dehydroepiandrosterone (DHEA) and its metabolite dehydroepiandrosterone sulfate (DHEAS) are synthesized in the adrenal glands and in the brain (Baulieu and Robel 1998). In a series of studies, the neuroprotective, antioxidant, and anti-inflammatory profile of DHEA and DHEAS has been emerged (Maninger et al. 2009). These neuroprotective properties expressed by DHEA seem to be dependent on their ability to bind and activate both tyrosine kinase (Trk) and pan-neurotrophin p75 (p75^NTR^) receptors (Charalampopoulos et al. 2004; Lazaridis et al. 2011; Pediaditakis et al. 2015).

The implication of the DHEA in anxiety has been suggested (Eser et al. 2006). High serum DHEA levels have been observed in patients suffering from panic (Tait et al. 2002) and PTSD (Rasmusson et al. 2004; Spivak et al. 2000). Interestingly, it has been proposed that this increase of DHEA and DHEAS concentrations, in response to adrenocorticotropic hormone (ACTH) stimulation, appears to be critical for the attenuation of the anxiety symptom and may be regarded as a compensatory response to stress (Rasmusson et al. 2010). In addition, treatment with DHEA was found to improve anxiety symptoms in schizophrenia patients (Stros et al. 2003). Preclinical findings also corroborate for a role of DHEA in anxiety. It has been reported that administration of DHEA induced an anti-anxiety-like behavior in rodents revealed in unconditioned exploration-driven anxiety tests such as the elevated plus maze (EPM) and the open field (OF) tests (Fedotova and Sapronov 2004; Maayan et al. 2006; Melchior and Ritzmann 1994). There is scarce evidence, however, whether DHEA could display an anti-anxiety-like effect in a conditioned non-exploration driven model of anxiety. Finally, it cannot be underestimated the serious undesired endocrine effects (hormone-dependent neoplasias) due to DHEA’s ability to be metabolized into estrogens, androgens, and progestins (Klinge et al. 2018; Webb et al. 2006). Based on the above, the potential clinical utilization of DHEA appears problematic.

BNN27 is a new synthetic derivative of DHEA, which, unlike DHEA, lacks androgenic or estrogenic undesired action since it does not activate steroid hormone receptor (Calogeropoulou et al. 2009). BNN27 is a small lipophilic compound, well tolerated, which crosses the blood brain barrier (BBB) (Bennet et al. 2016). In contrast to DHEA, BNN27 presents a high affinity for the TrkA and p75^NTR^ receptors of nerve growth factor (NGF) but does not affect pain thresholds (Pediaditakis et al. 2015; 2016a, b).

In a series of studies, BNN27’s antiapoptotic, anti-inflammatory, and antioxidant properties have been evidenced. Specifically, BNN27 was found to protect the PC12 cell line against serum deprivation–induced apoptosis at nanomolar concentrations (Calogeropoulou et al. 2009) and rescued from apoptosis TrkA-positive sympathetic sensory neurons and p75^NTR^-expressing TrkA-negative cerebellar granule neurons (Pediaditakis et al. 2016a). In addition, BNN27 reduced the pro-inflammatory factors, tumor necrosis factor alpha (TNFα), and interleukin-1 beta (IL-1β) while it increased the anti-inflammatory (IL-10 and Il-4) cytokine levels (Glajch et al. 2016). BNN27 was shown to attenuate the loss of motor neurons co-cultured with astrocytes derived from amyotrophic lateral sclerosis (ALS) patients with superoxide dismutase (SOD) mutations via the reduction of oxidative stress (Iban-Arias et al. 2018). BNN27 (30 and 90 mg/kg) reduced locomotor activity and exploration in rats but, when it administered at 30 mg/kg, did not affect animals’ performance in the light/dark (L/D) and forced swimming (FS) tests which are procedures measuring anxiety-like and depression-like behavior, respectively, in rodents (Kokras et al. 2020). On the contrary, acute challenge with a low dose range (3 and 6 mg/kg) of BNN 27 counteracted behavioral deficits, including cognition impairments, revealed either in glutamatergic or dopaminergic models of schizophrenia in rats (Pitsikas et al. 2021; Zoupa et al. 2019).

In this context, it is important to emphasize that the outcome of clinical and preclinical research suggests that oxidative stress and inflammation are involved in the pathogenesis of anxiety disorders (Michopoulos et al. 2017; Salim 2014; Smaga et al. 2015). Cognitive impairments are also observed in anxiety patients (Gkintoni and Ortiz 2023; Gulpers et al. 2022; Yang et al. 2015). Moreover, it is well documented that anxiety disorders are a typical feature in schizophrenia patients (for review see Braga et al. 2013).

Up to now, there is no information whether acute exposure to a low dosage of BNN27 (3 and 6 mg/kg) which exerted a beneficial effect in animal models resembling schizophrenia could induce anti-anxiety-like behavior in rats. The present study was designed aiming to elucidate this issue. To this end, the L/D (Crawley and Goodwin 1980); the OF (Prut and Belzung 2003), which are both unconditioned exploration-driven models of anxiety (Bouwknecht and Paylor 2008); and the contextual fear conditioning (CFC), which is a conditioned non-exploration driven model of anxiety (Resstel et al. 2006), tests were used. Finally, the ability of BNN27 to attenuate compulsive behavior (excessive self-grooming) induced by the serotonin 5-HT_2c_ receptor agonist (mCPP) (Bagdy et al. 1992) was also assessed.

## Materials and methods

### Subjects

Independent groups of naïve 3-month-old male Wistar rats (144 animals) (Hellenic Pasteur Institute, Athens, Greece) weighing 250–300 g were used. The animals were housed in Makrolon cages (47.5 cm length × 20.5 cm height × 27 cm width), three per cage, in a regulated environment (21 ± 1 °C; 50–55% relative humidity; 12-h/12-h light/dark cycle, lights on at 07.00 h) with free access to standard laboratory diet (pellets) for rats and water.

The procedures that involved animals and their care were conducted in conformity with the international guidelines in compliance with international guidelines and national (Animal Act, P.D. 160/91) and international laws and policies (EU Directive 2010/63). Experiments were approved by the local committee (Prefecture of Larissa, Greece, protocol number 386501/2023). Every effort was made to minimize the number of animals used and their suffering.

### Behavior

#### Experimental protocol

Experiments were conducted between 10.00 and 14.00 h in a room where only these animals were housed. Different populations of rats were used across different experiments. Each rat was tested only once. On the test day, the rats were transported to the test room and left in their home cages undisturbed for 2 h. To avoid the presence of olfactory cues, all the apparatuses were thoroughly cleaned with 20% ethanol and then wiped with dry paper after each trial. Animals’ behavior was video recorded. Data evaluation (of all four experiments except motility data of experiment 4) was subsequently performed using a stopwatch by experimenters who were unaware of the pharmacological treatment of each subject. Motor activity data evaluation of the experiment 4 was provided automatically from the test apparatus.

#### Drugs

All solutions were freshly prepared on the day of testing and were administered intraperitoneally (i.p.) in a volume of 1 ml/kg. BNN27 [(20R)-3*β*,21-dihydroxy-17*R*,20-epoxy-5-pregnene] was synthesized at the National Research Foundation (Calogeropoulou et al. 2009). BNN27 was suspended in saline (NaCl 0.9%) containing 0.1% Tween 80 and was sonicated for 5 min. mCPP (1-(3-chlorophenyl)piperazine) (Sigma, St. Louis, MO, USA) was dissolved in saline. Control animals received isovolumetric amounts of the specific vehicle solution used in each study. mCPP dose (0.6 mg/kg) was selected based on prior findings which this dose was found to cause excessive self-grooming (Graf et al. 2003; Peristeri and Pitsikas 2022).

#### Light/dark (L/D) test

The L/D box apparatus consisted of a wooden box (48 cm length × 24 cm height × 27 cm width) divided into two equal size compartments by a barrier that contained a doorway (10 cm height × 10 cm width). One of the compartments was painted black and was covered with a lid, and the other compartment was painted white and illuminated with a 60-W light bulb (Merlo Pich and Samanin 1989) positioned 40 cm above the upper edge of the box. The test was performed as described previously (Grivas et al. 2013). The animals were placed in the middle of the lit compartment, facing away from the dark chamber. The rats were allowed to freely explore the apparatus for 5 min. The observed variables were (a) the latency to enter (with all four paws) the dark compartment, (b) the number of transitions between the two compartments, and (c) the time spent in the light and dark compartments.

#### Open field (OF) test

The test apparatus consisted of an open box made of PPLEXIGLAS (70 cm length × 50 cm height × 70 cm width). The open field arena was divided by black lines into 16 squares of 17.5 × 17.5 cm^2^. The central four squares were defined as the central zone, in which animals’ activity was regarded as a measure of anxiety (Prut and Belzung 2003). The test was performed as described previously (Grivas et al. 2013). On the test day, each animal was then placed in the same corner of the open field arena and its behavior was recorded for 5 min. The observed variables were (a) the amount of the time spent in the central zone of the open field arena as defined by all forepaws being in the central four squares of the apparatus, (b) the number of squares crossed (i.e., horizontal activity), (c) the number of rearing behaviors (i.e., vertical activity, defined as raising both forepaws above the floor while balancing on hind limbs), and (d) the duration of grooming events.

#### Contextual fear conditioning (CFC) test

The apparatus consisted of a box made of PLEXIGLAS (50 cm length × 50 cm height × 50 cm width) with a grid floor composed of 17 stainless steel rods (3 mm in diameter). Electric shocks were delivered to the grid floor by an isolated electric shock generator.

For assessing in animals’ freezing behavior, a procedure adapted from a previous study was utilized (Gravious et al. 2006). On day 1, rats were placed individually into the chamber and received a single 2-min habituation trial. On day 2, the contextual conditioning trial was conducted. Rats were placed again individually into the apparatus, and after a 5-min period of acclimatization, three electric foot shocks (0.5 mA, 1 s) (Gravious et al. 2006) were delivered. The interval between the electric shocks was 1 min. One minute following the last foot shock, animals were removed from the apparatus and returned to their home cages. Testing was carried out 24 h after contextual conditioning, on day 3. The animals were again placed individually into the apparatus, and their freezing behavior (total amount of time) was recorded for 5 min. Freezing was defined as the total absence of body movements except for movement related to respiration.

#### Self-grooming behavior

Rats’ grooming behavior was assessed in an activity cage (catalog number 7420, Ugo Basile, Varese, Italy). The apparatus consisted of a box made of PLEXIGLAS (41 cm length × 33 cm height × 41 cm width). Every movement of the rat produced a signal caused by vibrations in the inductance and capacitance of resonance circuitry of the apparatus. The signals were then automatically converted into numbers that reflected horizontal activity counts. Changes in activity counts represent a standard behavioral assay for testing the motoric effects of drugs. For evaluating in rats’ grooming behavior, a procedure modified from previous studies was used (Graf et al. 2002; Peristeri and Pitsikas 2022). On day 1, rats received a single 10-min habituation session in the apparatus. On day 2, following appropriate treatment, animals were placed again into the apparatus, and the duration of grooming events was recorded for 20 min. Vibrations (unusual spontaneous behaviors); the nose, face, and head wash; body grooming; scratching; paw licking; head shaking; tail and genital grooming were considered components of grooming behavior (Graf et al. 2003). Further, locomotor activity, expressed as total counts over 20 min and number of rearing episodes (i.e., defined as raising both forepaws above the floor while balancing on hind limbs), were recorded.

#### Experiments 1, 2, and 3: effects of acute administration of low doses of BNN27 on rats’ performance in the L/D OF and CFC tests

Animals were randomly divided into four experimental groups with eight rats per group as follows: vehicle, BNN27 1 mg/kg, BNN27 3 mg/kg, and BNN27 6 mg/kg. To examine the effects of acute treatment with BNN27 on rats’ performance in the L/D, OF, and CFC tests, rats received a single injection of different doses of BNN27 or vehicle 40 min before testing (Pitsikas et al. 2021). Concerning the CFC test, vehicle and the different doses of BNN27 were injected on day 3, 40 min before testing (Jacob et al. 2009).

#### Experiment 4: effects of acute administration of low doses of BNN27 in counteracting mCPP-induced excessive self-grooming

Rats were randomly divided into six experimental groups (eight rats per group) as follows: vehicle + vehicle, vehicle + BNN27 3 mg/kg, vehicle + BNN27 6 mg/kg, mCPP 0.6 mg/kg + vehicle, mCPP 0.6 mg/kg + BNN27 3 mg/kg, and mCPP 0.6 mg/kg + BNN27 6 mg/kg. BNN27 and mCPP were administered 40 and 10 min, respectively, before testing (Graf et al. 2003; Pitsikas et al. 2021). Control animals received the respective vehicles 40 and 10 min, respectively, before testing.

### Statistical analysis

Data from experiments 1, 2, and 3 were expressed as mean ± SEM and were analyzed using one-way analysis of variance (ANOVA) test. The factor was treatment. Post hoc comparisons between treatment means were made using the Tukey’s *t* test. Self-grooming duration data from experiment 4 were not normally distributed (Shapiro–Wilk normality test failed, *p* < 0.05). Therefore, these data were expressed as medians and interquartile ranges and were analyzed using the Kruskal–Wallis non-parametric test. Post hoc pairwise multiple comparisons were made using the Newman-Keuls test. The other data from experiment 4 (locomotor activity and number of rearings) were expressed as mean ± SEM and were analyzed utilizing the two-way ANOVA test. The factors were mCPP and BNN27. A *p* value of < 0.05 was considered significant (Kirk 1968).

In all experiments, variances were homogeneous, and data were normally distributed (except self-grooming duration data of experiment 4).

## Results

### Experiment 1: effects of acute administration of low doses of BNN27 on rats’ performance in the L/D test

**Fig. 1:**
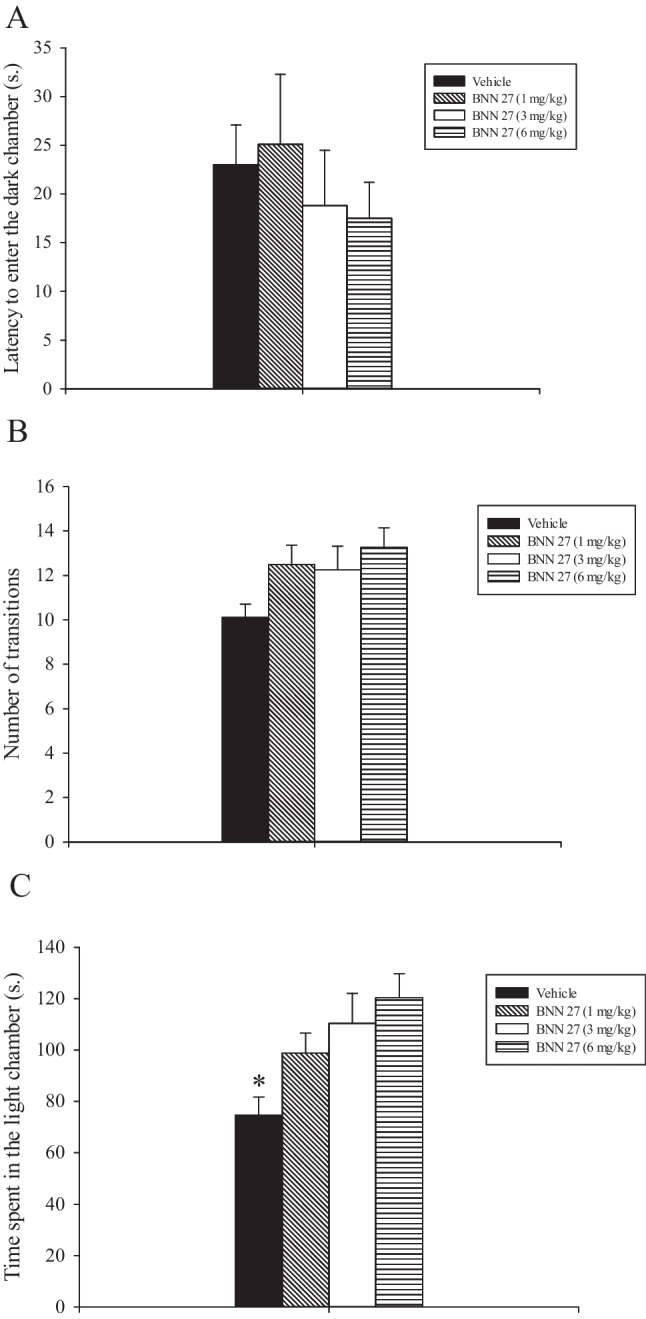
Light/dark test. Vehicle and BNN27 were injected i.p., 40 min before testing. Results are expressed as mean ± SEM. **A** Latency to enter the dark chamber. **B** Number of transitions. **C** Time spent in the light chamber. *p < 0.05 vs. all the other groups

Treatment with BNN27 did not affect the first entry into to the dark chamber (*F*_3, 31_ = 0.433, *p* = 0.702, not significant (n.s)) (Fig. 1A) and the number of transitions between the two compartments (*F*_3, 31_ = 2.187, *p* = 0.112, n.s) (Fig. 1B). Interestingly, BNN27 significantly increased the total time spent in the light chamber as revealed by a statistically significant effect of treatment (*F*_3, 31_ = 4.640, *p* = 0.009). The post hoc analysis conducted on these data showed that the vehicle-treated rats spent significantly less time in the lit chamber compared to their counterparts treated either with 3 or 6 mg/kg BNN27 (*p* < 0.05; Fig. 1C).

### Experiment 2: effects of acute administration of low doses of BNN27 on rats’ performance in the OF test

The effects of treatment with BNN27 on animals’ performance in the OF test are illustrated in Table 1. BNN27 did not affect the number of squares crossed (*F*_3, 31_ = 1.723, *p* = 0.185; n.s) and the rearing episodes (*F*_3, 31_ = 2.557, *p* = 0.075; n.s). BNN27 appeared to reduce grooming duration, but this effect did not reach a statistical significance (*F*_3, 31_ = 2.727, *p* = 0.063, n.s). By contrast, treatment with BNN27 increased the time spent in the central zone of the apparatus (*F*_3, 31_ = 3.638, *p* = 0.025). The post hoc comparisons showed that rats treated with 3 and 6 mg/kg BNN27 spent more time in the central zone of the OF apparatus compared to their vehicle-treated cohorts (*p* < 0.05).

**Table 1:** Effects of acute treatment with BNN 27 on rats’ performance in the open field test

Treatment	Number of squares crossed	Number of rearings	Time spent in the central zone (s)	Grooming duration (s)
Vehicle	86.1 ± 3.3	32.3 ± 0.6	6.4 ± 2*	12.4 ± 2.7
BNN 27 (1 mg/kg)	98.4 ± 2.9	32.5 ± 0.7	9 ± 1.1	6.8 ± 2.3
BNN 27 (3 mg/kg)	94 ± 5.4	35.6 ± 1.1	14.5 ± 2.4	7.3 ± 1.3
BNN 27 (6 mg/kg)	99 ± 5.7	35.3 ± 1.7	14.3 ± 2.6	4.9 ± 1.1

Data are expressed as mean ± SEM. of eight rats per treatment group. Vehicle and BNN 27 were injected intraperitoneally 40 min before testing

*p < 0.05 vs. the BNN27 3 and 6 mg/kg groups

### Experiments 3: effects of acute administration of low doses of BNN27 on rats’ performance in the CFC test

**Fig. 2:**
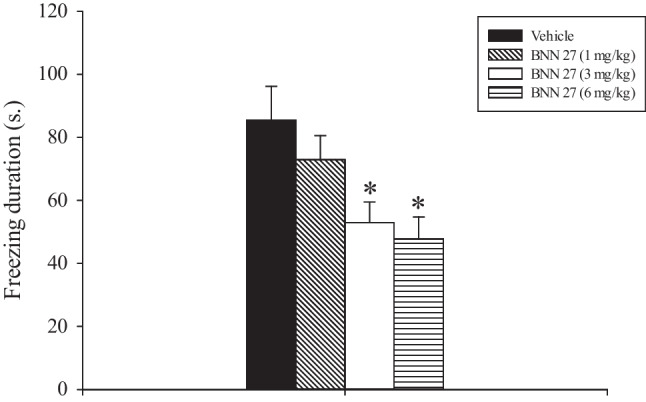
Contextual fear conditioning test. Vehicle and BNN27 were injected i.p., 40 min before testing. Results are expressed as mean ± SEM. *p < 0.05 vs. all the other groups

The effects of acute challenge with BNN27 on animals’ performance in the CFC test are depicted in Fig. 2. Treatment with BNN27 significantly reduced freezing duration (*F*_3, 31_ = 4.787, *p* = 0.008). The post hoc analysis conducted on these data showed that freezing levels of rats treated with 3 and 6 mg/kg BNN27 were significantly lower as compared to those expressed by the vehicle-treated animals (*p* < 0.05).

### Experiment 4: effects of acute administration of low doses of BNN27 in counteracting mCPP-induced excessive self-grooming

Statistical analyses of self-grooming duration data showed an statistically significant effect of treatment: *H*_(5)_ = 19.977,* p* = 0.001. These results indicate that rats receiving mCPP plus vehicle displayed higher self-grooming in comparison to all the other experimental groups including the mCPP + BNN27 3 mg/kg and the mCPP + BNN27 6 mg/kg-treated animals (*p* < 0.05; Table 2).

**Table 2:** Effects of acute treatment with BNN27 on excessive self-grooming induced by mCPP

Treatment	Grooming duration (s)
Vehicle + vehicle	116.5 (99.75–146.25)
Vehicle + BNN27 (3 mg/kg)	111.5 (91.75–146)
Vehicle + BNN27 (6 mg/kg)	104 (65.25–136.5)
mCPP (0.6 mg/kg) + vehicle	236.5 (174.75–282.5)*
mCPP + BNN27 (3 mg/kg)	174.5 (147.75–197.5)
mCPP + BNN27 (6 mg/kg)	189.5 (122.75–210.5)

Data are expressed as medians and interquartile ranges of eight rats per treatment group. mCPP and BNN 27 were injected intraperitoneally 40 and 10 min, respectively, before testing

* p < 0.05 vs. all the other groups

**Fig. 3:**
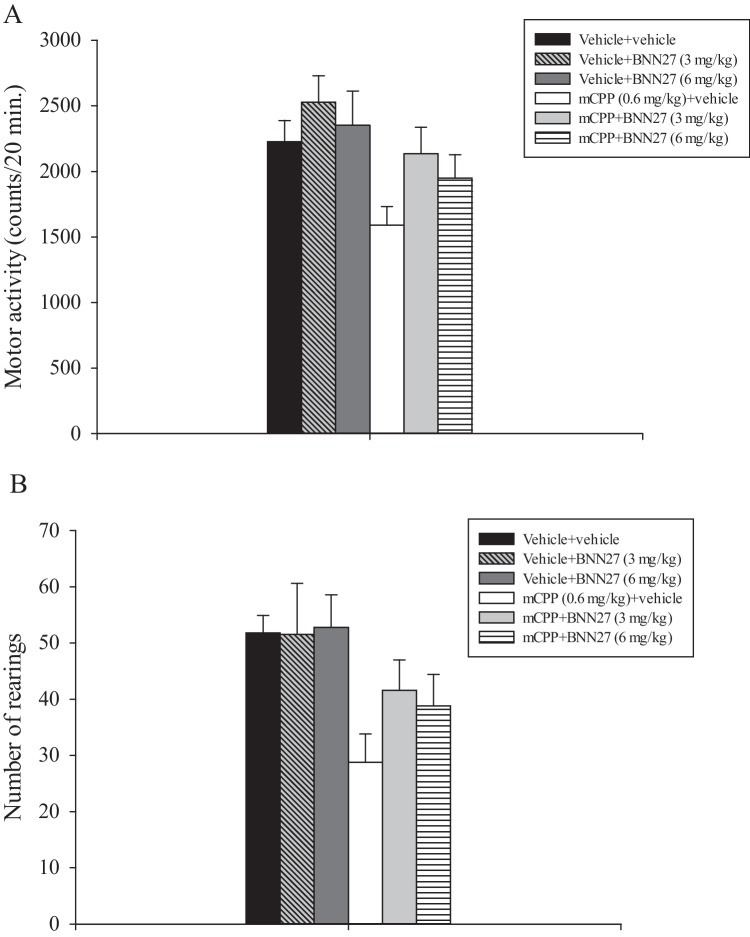
Self-grooming test. Vehicle, mCPP, and BNN27 were injected i.p., 40 min and 10 min, respectively, before testing. Results are expressed as mean ± SEM. **A** Locomotor activity. **B** Number of rearings

A two-way ANOVA conducted on motility data revealed a significant main effect of mCPP (*F*_1, 47_ = 8.962, *p* < 0.005), but not of BNN27 (*F*_2, 47_ = 2.369, *p* < 0.106, n.s.), or a significant interaction between mCPP and BNN27 (*F*_2, 47_ = 0.247, *p* < 0.783, n.s.; Fig. 3A). These results indicate that all groups of rats receiving mCPP displayed lower motility levels respect to all the other experimental groups. Post hoc comparisons between treatment means were not conducted since a significant interaction between mCPP and BNN27 was not reached (Kirk 1968).

Analysis of rearing episodes’ data evidenced a main effect of mCPP (*F*_1, 47_ = 10.456, *p* < 0.002), but not of BNN27 (*F*_2, 47_ = 0.673, *p* = 0.515, n.s.), or a significant interaction between mCPP and BNN27 (*F*_2, 47_ = 0.643, *p* = 0.531, n.s.; Fig. 3B). These findings suggest that all groups of rats receiving mCPP displayed lower number of rearing episodes as compared to all the other experimental groups. Post hoc pairwise multiple comparisons between treatment means were not performed since a significant interaction between mCPP and BNN27 was not achieved (Kirk 1968).

## Discussion

L/D test has been shown to reliably predict the anxiolytic- and anxiogenic-like effects of drugs in rodents. L/D test is a procedure that is based on the innate aversion of rodents for highly illuminated areas and on their spontaneous exploratory behavior in response to mild stressors that is a novel environment and light (Crawley and Goodwin 1980). This test has the advantages of being quick and easy to use without prior training of the animals and neither food nor water deprivation is required (Bourin and Hascoet 2003). Transitions in this test are considered an index of activity/exploration because habituation over time is seen with this measure, whereas the time spent in each chamber reflects aversion/attraction (Belzung et al. 1987). Acute administration of 3 and 6 but not 1 mg/kg BNN27 to rats increased the time spent in the light chamber of the L/D box compared to their vehicle-treated cohorts. Further, BNN27 did not influence both the latency of the first entry into the dark chamber of the apparatus and the number of transitions between the two compartments of the apparatus.

OF test is a standard neophobic test of anxiety. It involves encounter with a novel environment and give rise to behavioral and physiological reactions related to anxiety. In this test, rodents usually tend to avoid open spaces. Thus, the time spent in the central area of an open field arena is a measure of an anxiety state (Prut and Belzung 2003). A single injection of BNN27 (3 and 6 but not 1 mg/kg) significantly augmented the time spent by rats in the central zone of the OF apparatus.

Exposure to a novel environment increases also self-grooming tendencies (Jolles et al. 1979). Self-grooming is a congenital rodent behavior characterized by a sequential pattern of movements, reflects compulsive behavior, and is considered an anxiety index (Estanislau et al. 2019). BNN27 at 6 mg/kg seemed to reduce grooming activity, but this effect did not reach a statistical significance.

CFC test is considered a preclinical model of anxiety (e.g., Biojone et al. 2011; Jacobs et al. 2009; Krystal et al. 2012; Yan et al. 2016) and might resemble PTSD symptoms (e.g., freezing) (Bertaina-Anglade et al. 2017; Hooversmith et al. 2019; Torok et al. 2019). PTSD is a major anxiety disorder that may develop after an individual has experienced or witnessed a severe traumatic event (Torok et al. 2019). CFC measures fear, in terms of freezing, linked to a context where foot shock occurred (Pain et al. 2002; Resstel et al. 2006). BNN27 (3 and 6 but not 1 mg/kg) was found efficacious in attenuating freezing behavior.

The above reported findings cannot be attributed to a potential effect of BNN27 on motility since the number of transitions between the two different chambers of the L/D box, the number of squares crossed and the number of rearings recorded in the OF were not dissimilar among the different treatment groups. An unspecific motoric effect of BNN27 on rats’ performance in the CFC can also be ruled out since at this low dose range, BNN27 did not modify animals’ motor activity (Pitsikas et al. 2021) while reduction of it has been observed at a high dose of BNN27 (90 mg/kg) (Kokras et al. 2020).

The anxiogenic properties of the selective 5-HT_2c_ receptor agonist mCPP are evidenced in either preclinical or clinical studies (e.g., Charney et al. 1987; Singewald et al. 2003). Further, it has been reported that mCPP exaggerates self-grooming in rats (Bagdy et al. 1992; Graf et al. 2003; Peristeri and Pitsikas 2022).

In agreement with prior results (Bagdy et al. 1992; Graf et al. 2003; Peristeri and Pitsikas 2022), acute exposure to mCPP (0.6 mg/kg) significantly raised up self-grooming activity in rats and reduced both horizontal motor activity and number of rearings. Acute administration of BNN27 (3 and 6 mg/kg) attenuated excessive self-grooming caused by mCPP. BNN27, at any dose tested, did not affect grooming activity in control animals. BNN27 appeared to counteract the effects of mCPP on horizontal (hypomotility) and vertical activity (decrease of rearings), but this action did not achieve a statistical significance. These latter results suggest that the effects of BNN27 on self-grooming might be unrelated to its action on parameters reflecting physical activity.

Although mCPP displays affinity for the family of the 5-HT_2_ receptors, its action on self-grooming has been shown to be mediated by the 5HT_2c_ receptor (Graf et al. 2003). The 5-HT_2c_ receptor is located in brain regions critically involved in anxiety and OCD, including the basal ganglia and orbitofrontal and cingulate cortices (Clemmet et al. 2000; Pasqualetti et al. 1999; Pompeiano et al. 1994; Santana and Artigas 2017). Accordingly, we hypothesize that BNN27 might counteract the effects of mCPP on self-grooming by an antagonistic action at the 5-HT_2c_ receptor site. Additional research is required to elucidate this point.

Summarizing, the present results indicate that BNN27, like DHEA, expressed an anti-anxiety action in unconditioned exploration-driven procedures that are based on the conflict between the desire to explore and avoidance of novel spaces as are the L/D and OF tests. Moreover, BNN27 displayed an anxiolytic effect in a conditioned non-exploration driven model of anxiety (Bouwknecht and Paylor 2008) such as the CFC test. Interestingly, the effective anti-anxiety doses of BNN27 (3 and 6 mg/kg) are the same that exerted beneficial actions in animal models of cognition (Pitsikas and Gravanis 2017) and schizophrenia (Pitsikas et al. 2021; Zoupa et al. 2019). BNN27 administered acutely at 1 mg/kg did not express any appreciable biological activity.

Results of the present study are in partial contrast with previous work in which a higher dose range of BNN27 than that used in our study was found to suppress motility (90 mg/kg) and exploration (30 and 90 mg/kg). Additionally, BNN27 (30 mg/kg) did not affect rats’ performance in procedures reflecting anxiety (L/D test) or depression (FS test). Further, a lower dose of BNN27 (10 mg/kg) did not influence motility, exploration, and did not have an impact on rats’ performance in the L/D and FST tests (Kokras et al. 2020).

Overall, the anxiolytic effects of BNN27 were observed at 3 and 6 mg/kg but not at the “side” doses of 1, 10, 30, and 90 mg/kg. This pattern of results suggests that a bell-shaped dose–effect curve might underlie BNN27’s biological effects. At present, the biological bases of the bell-shaped dose–response curves are unknown, although receptor fatigue or tachyphylaxis (Day 1979) has been proposed as potential mechanisms (Martinez 1986). Higher doses of BNN27 may have two repercussions: (a) activate other receptors with lower affinity compared to TrkA/p75^NTR^ receptors, which might counteract its anti-anxiety effects, and (b) provoke desensitization through internalization of its TrkA/p75^NTR^ receptors and their known long-lasting turnover and their intracellular trapping (Pediaditakis et al. 2016a).

The mechanism(s) of action by which BNN27 might exert its anti-anxiety effects is still under investigation. Research is required to elucidate the exact role of BNN27 in anxiety. In this context, it has been recently shown that a low dose (10 mg/kg) of BNN27 was able to increase GABA concentrations in the hippocampus in either male or female rats. It has been suggested that an increase of GABA levels in hippocampus might be correlated with a potential anxiolytic activity (Holm et al. 2011). This latter observation might provide a support for the anti-anxiety-like behavior expressed by BNN27 revealed in the present study. Recent evidence indicates that deletion of p75^NTR^ receptors in mice leads to physiological and morphological changes in the amygdala and altered anxiety behavior that is linked to the limbic system (Busch et al. 2017; Puschban et al. 2016). It is thus possible that BNN27 might affect anxiety through its interaction with p75^NTR^ receptors and cross talk with the 5-HT_2_ or GABA_A_ receptors strongly involved in anxiety circuits (Pediaditakis et al. 2016b).

Moreover, the potent antioxidant and anti-inflammatory properties of BNN27 evidenced in different studies and described above might also provide an alternative explanation of the present findings.

The current study presents some limitations. The effects of BNN27 were shown following acute treatment solely in behavioral studies conducted exclusively in male rats. Importantly, it is well documented that the prevalence of anxiety for females is roughly twice that for males (Kessler et al. 2012).

Additional research, therefore, is mandatory to definitively establish the efficacy of BNN27 as an anxiolytic agent. The investigation of the potential anti-anxiety-like action of BNN27 on both male and female rodents across a large variety of behavioral paradigms might be of high translational value. Further, treatment strategies should include either acute or prolonged administration of the compound. Biochemical, molecular, and electrophysiological studies should also be conducted aiming to provide a solid support to the here presented behavioral results.

In summary, the present findings indicate that the DHEA-synthetic derivative BNN27 which devoid of the undesired endocrine effects of DHEA expressed an anti-anxiety-like behavior revealed in a battery of behavioral procedures resembling different subtypes of anxiety disorders. The current results, although preliminary, offer a new lead molecule, BNN27, to develop new therapeutic agents for the treatment of anxiety.

## Data Availability

The data that support the findings of this study are available upon request from the corresponding author.

## Declarations

The authors declare that the experiments comply with the current laws of Greece.
